# Preoperative Cognitive Impairment and the Prevalence of Postoperative Delirium in Elderly Cancer Patients—A Prospective Observational Study

**DOI:** 10.3390/diagnostics11020275

**Published:** 2021-02-10

**Authors:** Anca Irina Ristescu, Georgiana Pintilie, Mihaela Moscalu, Daniel Rusu, Ioana Grigoras

**Affiliations:** 1 Department of Anaesthesia and Intensive Care, School of Medicine, Grigore T. Popa University of Medicine and Pharmacy, 700115 Iasi, Romania; anca.ristescu@umfiasi.ro (A.I.R.); pintilie.g.georgiana@email.umfiasi.ro (G.P.); ioana.grigoras@umfiasi.ro (I.G.); 2 Department of Anaesthesia and Intensive Care, Regional Institute of Oncology, 700483 Iasi, Romania; daniel-mihai.m.rusu@d.umfiasi.ro; 3 Department of Preventive Medicine and Interdisciplinarity, Grigore T. Popa University of Medicine and Pharmacy, 700115 Iasi, Romania

**Keywords:** preoperative cognitive impairment, postoperative delirium, elderly patients, Mini-Cog test, Nu-DESC score

## Abstract

Preoperative cognitive impairment (PCI) in cancer patients includes a broad spectrum of neurocognitive changes produced by complex interplay of patient, tumoural and treatment-related factors. Reduced preoperative cognitive reserve can favour the emergence of postoperative delirium (POD). The study aims to document PCI prevalence and to assess the relationship with POD in elderly cancer patients. The prospective observational study included consecutive patients scheduled for elective surgery; PCI was assessed with Mini-Cog test and defined at a score ≤ 3, POD was screened using Nursing Delirium Screening Scale (Nu-DESC) and defined at a score ≥ 2. Data on education, American Society of Anesthesiologists (ASA) score, preoperative medications, substance use, comorbidities, sensorial deficits, surgery and anaesthesia type, anaesthetic drugs, Mini-Cog score, postoperative pain, Nu-DESC were collected. In total, 131 patients were enrolled, mean age 72.1 ± 5.9 years. PCI prevalence was 51.9% (*n* = 68). POD prevalence was 19.8% (*n* = 26), with significantly higher value in PCI patients (27.9% vs. 11.1%, *p* = 0.016). In multivariate analysis, Mini-Cog score ≤ 3 (OR = 2.6, *p* = 0.027), clock draw (OR: 2.9, *p* = 0.013), preoperative renal dysfunction (OR = 2.6, *p* = 0.012), morphine (OR = 2.7, *p* = 0.007), metoclopramide (OR = 6.6, *p* = 0.006), and high pain score (OR = 1.8, *p* = 0.018) had a significant association with POD development. In this sample of elderly patients, PCI had a high prevalence and predicted the emergence of POD. Incorporating Mini-Cog test into the preoperative evaluation of onco-geriatric patients seems valuable and feasible.

## 1. Introduction

The number of elderly cancer patients undergoing diagnostic, curative, supportive or palliative surgical interventions is significantly increasing. Older adults (aged 65 years and more) are currently the fastest-growing segment of the population in many countries around the world and the number is expected to further increase. In Romania, the percentage of elderly persons is expected to double by 2050 [1]. At the same time, cancer incidence in elderly patients is projected to increase with 67% by 2030, generating a concomitant rise in the number of cancer surgeries [2,3]. Overall, it is estimated that, in 2030, out of 21.6 million new cancer cases, about 17.3 million will need surgery and 10 million of those patients will be from low- and middle-income countries [4]. 

Preoperative cognitive impairment (PCI) includes a broad spectrum of neurocognitive changes, varying from mild cognitive impairment to dementia, and consists of the decline of one or more key domains of the cognitive functions (memory, language, visuospatial, executive functioning, calculation) [5,6]. PCI can be the result of patient characteristics—cognitive reserve, genetics (polymorphism, epigenetics), comorbidities and chronic medication, of the primary disease—cancer type, site, stage, tumour inflammation, and of multimodal treatments [6]. Older adults with impaired cognition tend to have an increased rate of postoperative complications [7,8].

Perioperative period must be considered, for many reasons, a highly vulnerable time frame for all oncologic patients. The development of any postoperative complication can alter both surgical and oncological outcomes [9,10,11]. Especially in elderly patients, the complex association between the low functional reserve, cancer disease, and surgery aggressiveness generate a higher risk of postoperative complications and increased mortality. Accordingly, there is an urgent need to identify predictors of adverse outcomes in this age group. Preoperative risk stratification, consisting of a detailed evaluation of end-organ function, allows quantifying the postoperative risk. While vital organ functions (pulmonary, cardiac, renal) are commonly assessed before major surgery, cognitive reserve is only subjectively evaluated, without the routine use of an objective scale. Accumulating literature data supports the existence of a high rate of Perioperative Neurocognitive Disorders in elderly surgical cancer patients. 

Postoperative delirium (POD), currently defined as an acute and fluctuating disturbance in attention and awareness accompanied by cognitive dysfunction, is a common, serious, and potentially fatal disorder related to neuroinflammation and must be considered as an acute end-organ dysfunction [5,12,13]. Highly under-recognised and undiagnosed in elderly patients in the absence of routine monitoring, it has been shown to be potentially preventable in 30–40% of cases. In clinical studies, POD was associated with increased major postoperative complications, persistent neurocognitive disorder, longer hospital stay, higher medical costs, and increased mortality [12,14,15]. Cancer patients tend to have a greater risk for POD as a cumulative effect of cancer biology, chronic inflammation, neo-adjuvant treatment, nutritional deficits, stress of diagnosis, and treatment and pharmacologic interactions [16]. Preoperative identification of predisposing factors has a major role in POD prevention. 

In the current study we hypothesised that perioperative neurocognitive disorders are highly prevalent in older cancer patients and that the presence of PCI will be associated with the occurrence of POD. To test this hypothesis, we screened the cognitive function of elderly cancer patients with Mini-Cog test prior to elective surgery and we investigate the relationship of the Mini-Cog score to the development of POD.

## 2. Materials and Methods

### 2.1. Study Design

We performed a prospective observational cohort study conducted in the Anaesthesia and Intensive Care Department, Regional Institute of Oncology, Iasi, Romania. This was a hospital quality improvement project intended to introduce screening tools for PCI detection on Pre-Anaesthesia Consultation Clinic (PACC) and for POD diagnosis on Post-Anaesthesia Care Unit (PACU). The study protocol was approved by the local Clinical Research Ethics Committees and the informed consent was obtained from all patients.

### 2.2. Patients

Potential participants were screened on PACC during the preoperative evaluation. Inclusion criteria were age 65 years or older, cancer diagnosis (solid tumour), elective surgery, and postoperative admission to the PACU. Exclusion criteria were central nervous system (CNS) cancer or cerebral metastases, preoperative diagnosis of dementia, ASA score > 3, emergency or day surgery, and transfer to the ICU after surgery.

### 2.3. Anaesthesia and Perioperative Care

Patients received general or locoregional anaesthesia depending on surgical and patient-related characteristics and were not premedicated. Intraoperative monitoring of patients undergoing general anaesthesia included electrocardiogram, noninvasive blood pressure, oxygen saturation, end-tidal partial pressure of carbon dioxide, end-tidal concentration of sevoflurane, oesophageal temperature, and urine output. Invasive blood pressure and central venous pressure were measured when clinically indicated. The induction of general anaesthesia was performed intravenously with fentanyl 1–2 μg/kg, propofol 1–2 mg/kg, and rocuronium 0.6 mg/kg. Sevoflurane at a minimal alveolar concentration (MAC) of 0.8–1, fentanyl and rocuronium were used for the maintenance. Multimodal analgesia was started before the induction of anaesthesia and consisted of administration of acetaminophen, metamizole, nonsteroidal anti-inflammatory drugs, following the department protocol. Fluid management was at the discretion of the attending anaesthesiologist.

### 2.4. Data Collection

Perioperative variables considered risk factors for the development of POD were recorded. 

Preoperative data included patient characteristics (age, gender, body mass index), education level, substance use, sensorial deficits, chronic medication (e.g., benzodiazepines, barbiturates, nitrates, opiates, antidepressants, corticosteroids), presence of polypharmacy (≥3 drugs). Cognitive function was evaluated by a senior anaesthesiologist with Mini-Cog test. The presence of comorbidities (cerebrovascular, Parkinson’s diseases, depression, anxiety, sensorial deficits, cardiovascular, diabetes, anaemia, renal impairment) and their severity (Charlson Comorbidity Index), American Society of Anesthesiologists (ASA) score, type of cancer and plasma levels of sodium, glucose, urea, creatinine, and C Reactive Protein were also recorded. Anaemia was defined according to World Health Organisation as a haemoglobin value less than 12 g/dL in women and 13 g/dL in men.

Intraoperative data included the type and duration of surgery, type of anaesthesia (general, regional or local anaesthesia), anaesthetic drugs, estimated blood loss, type and volume of intravenous fluids. 

During the postoperative period, patients were screened for POD using Nu-DESC during the first 2 days after surgery. Collected data also included postoperative pain scored by numerical rating scale (NRS, ranging from 0 to 10) and PACU and hospital length of stay.

### 2.5. Neurocognitive Assessment

#### 2.5.1. Preoperative Cognitive Function 

The screening tool for the neuropsychological evaluation was Mini-Cog test [17] (Romanian translation) applied on PACC by a senior anaesthetist. This test consists of a three-item recall test for memory and a clock drawing test for visuo-spatial representation and executive function and is graded on a 5-point scale (Appendix A). Patients received 1 point for each word recalled and 2 points for a normal clock drawing. Normal cognition was considered for scores of 4 and 5, mild cognitive impairment for scores of 2 and 3, and severe cognitive impairment for a score less than 2. Mini-Cog test was previously validated in community-based populations, it has a high sensitivity (99%) and specificity (93%) for detecting cognitive impairment in older adults, and has a minimal education, language or ethnic bias [18]. Healthcare professionals can evaluate and score patients using this tool in 2–5 min after receiving proper training.

#### 2.5.2. Postoperative Delirium (POD)

Delirium was detected using the Nursing Delirium Screening Scale (Nu-DESC) [19] (Romanian translation). The Nu-DESC is a standardised tool for the diagnosis of POD, developed in 2005 by Gaudreau et al. based on the Confusion Rating Scale [19]. It was validated in both oncologic and PACU/postoperative patients (86% and 95% sensitivity and 87% specificity) and has been recommended by European Society of Anaesthesiology evidence-based and consensus-based guideline on postoperative delirium since 2017 [12,20,21]. Compared with other delirium screening tests, Nu-DESC implies a minor interaction with the patient, minimal healthcare training, is fast (takes under 2 min) and it can identify patients in the early/prodromal phase of this condition [22,23]. The Nu-DESC algorithm is based on the assessment of five main areas of POD: disorientation, inappropriate behaviour, inappropriate communication, illusions/hallucinations, and psychomotor retardation (Appendix B). Each item is scored based on severity with 0—absent, 1—mild, and 2—severe. Positive Nu-DESC is considered a score ≥2, with a maximum total value of 10. We screened elderly cancer patients on PACU and on the surgical wards three times a day for the first 2 days after surgery.

### 2.6. Outcomes

The primary endpoint was the prevalence and the severity of PCI, detected with Mini-Cog test in elderly cancer patients. Secondary end-points included (1) POD prevalence in older cancer patients, (2) the relationship between PCI and POD, and (3) identification of other perioperative predictors for POD.

### 2.7. Statistical Analysis

The SPSS 24.0 for Windows (SPSS Inc., Chicago, IL, USA) software was used for all statistical analyses. Continuous variables were presented as mean or median and were analysed using the Mann–Whitney U test. Categorical variables were analysed using the Pearson Chi-square test. Univariate and multivariate analysis of prognostic factors for delirium were performed and odds ratios (OR) were calculated by logistic regression analysis. The predictive power was evaluated based on the receiver operating characteristic (ROC) curve, taking into account the area under the curve (AUC). Tests were performed whenever appropriate, and *p*-values of less than 0.05 were considered of statistical significance.

The Joinpoint Regression program (version 4.8.0.1-22 April 2020; National Cancer Institute, Bethesda, MD, USA) was used to analyse crude rate trends. Thus, association patterns (segments) can be identified, and APC values (annual percentage changes) are estimated for each identified segment.

In the case of our study, the APC estimates (makes a prediction) the magnitude of the trend of percentage change in the frequency of postoperative delirium in the range of variation of the Mini-Cog score. A safe segment was identified (Mini-Cog score 0–5). Significance tests on the assessment of the change in the crude rate in each segment use a Monte Carlo permutation method. They allow testing whether an apparent change in the gross rate trend is statistically significant.

## 3. Results

### 3.1. Baseline Characteristics

**Figure 1:**
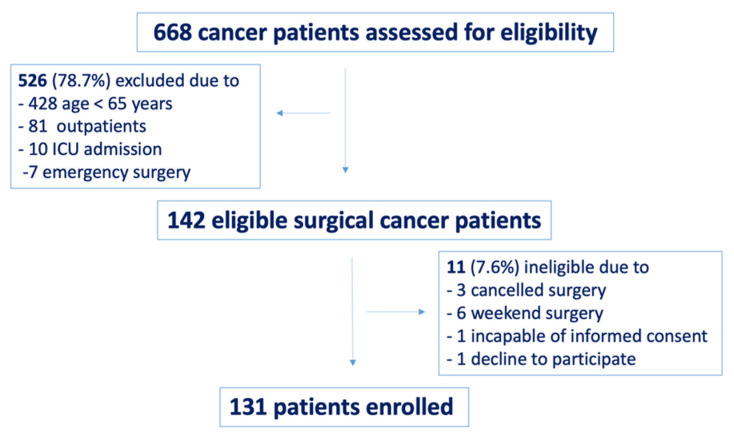
Study flow chart.

The flow of patients through the study is presented in Figure 1. Between January and April 2018, 668 surgical cancer patients were screened during the preanesthetic assessment; of these, 526 (78.7%) patients were excluded based on age, planned postoperative ICU admission, day or emergency surgery. In total, 142 patients were considered eligible for the study. Eleven (7.6%) patients had cancelled or week-end surgery, were incapable of informed consent or decline to participate. A total of 131 elderly cancer patients were finally enrolled and screened for cognitive impairment on PACC.

A total of 131 elderly cancer patients were preoperatively evaluated with Mini-Cog test. The mean age was 72.1 ± 5.9 years and 49.6% (*n* = 65) were female. More than half of patients had polypharmacy (58%) and 90% associated one or more comorbidities. The most common location of the primary tumour was gastro-intestinal (*n* = 62), followed by gynaecological (*n* = 21), genitourinary (*n* = 16), breast (*n* = 12), skin (*n* = 13), and lung cancer (*n* = 7) (Table 1). 

Baseline characteristics of the impaired and normal cognition groups were compared. The education level, alcohol consumption, sensorial deficits, comorbidities, ASA score, and the cancer type were similar in the two groups. The impaired cognition group was older (*p* = 0.001) and had a higher incidence of polypharmacy (*p* = 0.036) comparing to normal cognition group (Table 1).

### 3.2. Prevalence and Severity of PCI

**Figure 2:**
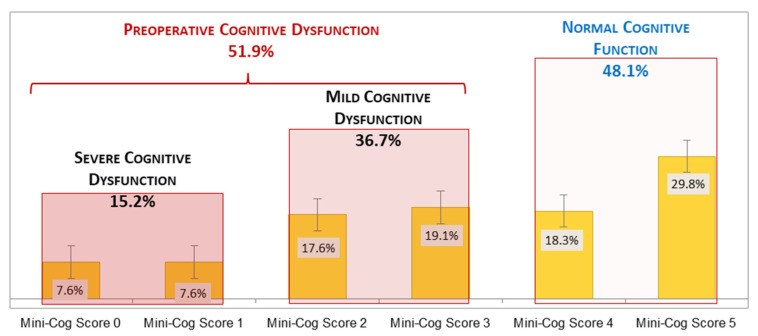
Preoperative cognitive function in elderly cancer patients.

The overall prevalence of impaired cognition at baseline was 51.9% (*n* = 68), with 15.2% (*n* = 20) severe (Mini-Cog score = 0–1) and 36.7% (*n* = 48) mild (Mini-Cog score = 2–3) cognitive impairment (Figure 2). The prevalence of cognitive impairment increased significantly (*p* = 0.001) with every decade of age, as follows: for 60–69 years was 38% (21 of 55 cases), for 70–79 years was 54% (30 of 56 cases), and for 80–89 years was 85% (17 of 20 cases). 

### 3.3. Postoperative Delirium Evaluation

The prevalence of postoperative delirium defined by Nu-DESC score ≥ 2, was 19.8% (*n* = 26). When we considered POD diagnosis at a value of Nu-DESC score ≥ 1 in order to increase test sensitivity, the prevalence of POD further increases to 38.9% (*n* = 51).

The age of patients with POD was significantly higher (*p* = 0.007) and all received general anaesthesia. 

A significant correlation was noted between ASA score and POD development (*p* = 0.037) (Nu-DESC ≥2—ASAIII: 69.2% vs. Nu-DESC <2—ASAIII: 46.6%), mentioning that patients with ASA score more than III were excluded. We did not find a positive correlation with sensorial deficits (*p* = 0.987), polypharmacy (*p* = 0.631), alcohol consumption (*p* = 0.365), or Charlson Comorbidities Index (*p* = 0.215) (Table 2). However, a significant correlation between cancer type and POD was found (*p* = 0.038), indicating an increased frequency of this postoperative complication in colorectal cancer. 

### 3.4. The Relationship between PCI and POD 

**Figure 3:**
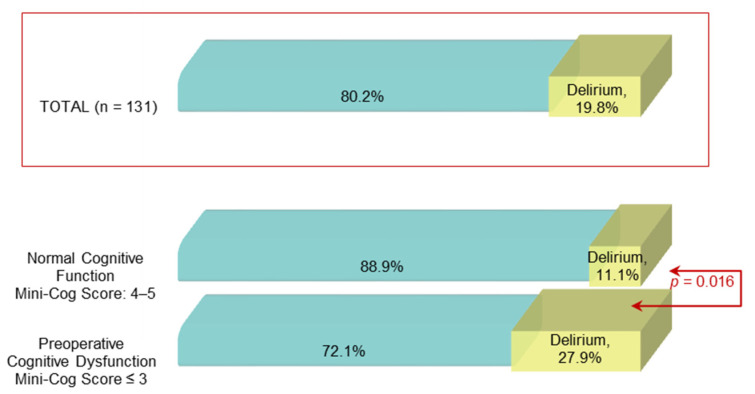
Postoperative delirium prevalence among patients with normal and impaired cognitive function.

POD occurred in 19 (27.9%) of 68 patients with preoperative impaired cognition (Mini-Cog score ≤3) and only in 7 (11.1%) of 63 patients with normal cognitive function (Mini-Cog score >3) (*p* = 0.016), as shown in Figure 3.

In patients diagnosed with PCI, we did not find a significant association between POD prevalence and age decades: 15.8% for 60–69 years (2 of 18 cases), 57.9% for 70–79 years (11 of 19 cases), and 26.3% for 80–89 years (5 of 12 cases) (*p* = 0.212).

**Figure 4:**
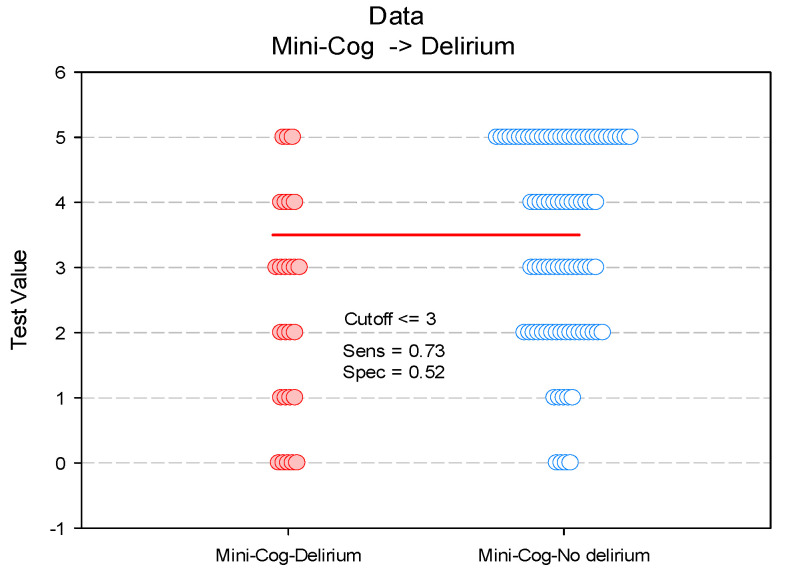
Paired histogram for estimating the cut-off value of the Mini-Cog score on predictability on POD.

We also investigated the Mini-Cog score cut-off value for the prediction of POD. For a Mini-Cog score cut-off point set at ≤ 3, the sensitivity of this test was 73% and the specificity 51% (Figure 4, Table 3).

A total of 51.9% of the patients (*n* = 61) had a risk score ≤ 3, of which 19 experienced delirium, with a positive predictive value of 60.2%. The negative predictive value was 65.8% (Table 3).

Based on the Joinpoint model, predicted of probability of delirium frequency was made according to the Mini-Cog score. Only one segment (linear model) was identified for the Mini-Cog score variation interval. A linear decrease in the probability of postoperative delirium cases for an increase in score values. 

**Figure 5:**
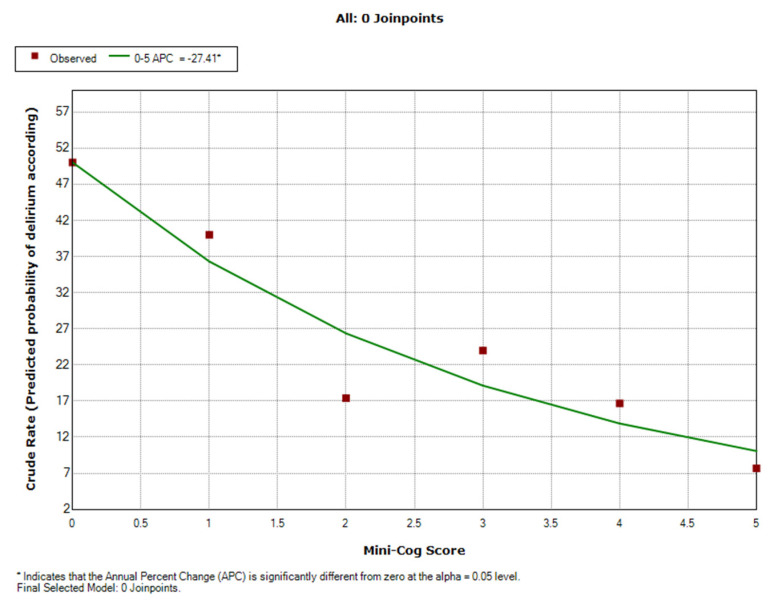
Scatter plot of the predicted probability of postoperative delirium according to preoperative cognitive impairment (Mini-Cog score).

Thus, the predicted probability for POD decreases significantly by 27.4% (APC = −27.4; 95%CI: −40.3–−11.7; *p* = 0.01) for the variation interval of the Mini-Cog score. (Table 4, Figure 5).

APC—percentage change in POD probability (%) for a one-unit increase in Mini-Cog score.

In our study for a Mini-Cog score between 0 and 1, the probability of postoperative delirium decreased from 50% to 37%. For the values of the Mini-Cog score between 1 and 3 the probability of postoperative delirium decreases from 37% to 20%, and for values higher than 3 the probability of POD decreases significantly, reaching a frequency of cases less than 10%.

### 3.5. Perioperative Predictors for POD

To identify predictors for POD in our cohort of elderly cancer patients, we performed an age-adjusted univariate and multivariate logistic regression analysis. As shown in Table 5, Mini-Cog score (OR = 2.6, CI 95%: 1.02–7.08, *p* = 0.006) and its both components, clock draw (OR = 2.9, CI 95%: 1.17–7.46, *p* = 0.021), and word recall (OR = 1.6, CI 95%: 1.11–4.28, *p* = 0.032), together with preoperative renal dysfunction (OR = 3.2, 95% CI: 1.25–8.25, *p* = 0.015), the type of surgery (OR = 2.6, CI 95%: 1.48–4.94, *p* = 0.023), duration of surgery (OR = 1.5, CI 95%: 1.26–3.51, *p* = 0.032), morphine use (OR = 3.3, CI 95%: 1.31–8.53, *p* = 0.011), metoclopramide use (OR = 5.8, CI 95%: 4.92–10.2, *p* = 0.041), and increased pain score (OR = 1.3, CI 95%: 1.05–1.6, *p* = 0.014) were significant factors for POD in the univariate analysis. Plasma levels of sodium, glucose, and C reactive protein, estimated blood loss, type and volume of intraoperative intravenous fluids were also recorded but not included in the final analysis, as their values were similar in both groups.

In multivariate analysis, Mini-Cog score (≤3) (OR = 2.6, 95% CI: 1.15–3.9, *p* = 0.027), clock draw (OR = 2.9, 95% CI: 1.17–9.64, *p* = 0.013), preoperative renal dysfunction (OR = 2.6, 95% CI: 1.85–8.06, *p* = 0.012,), morphine use (OR = 2.7, 95% CI: 1.89–8.30, *p* = 0.007), metoclopramide use (OR = 6.6, CI 95%: 2.82–13.42, *p* = 0.006), and high pain score (OR = 1.8, 95% CI: 1.07–2.64, *p* = 0.018) had a significant association with POD development (Table 5). 

## 4. Discussion

In the present study we found that preoperative cognitive impairment (PCI) assessed by Mini-Cog test screening was both highly prevalent in a cohort of geriatric patients undergoing elective surgery for solid cancer and predictive for the development of POD. 

In recent years, there has been a growing concern among elderly cancer patients and their families on the adverse consequences of medical or surgical oncologic treatment on the cognitive function. The vast majority of these patients are more interested in maintaining their memory and attention than in survival. From this perspective, detecting preoperative subclinical dysfunction and preventing postoperative acute neurocognitive deterioration are of paramount importance.

Cognitive function decline and impairment in elderly patients diagnosed with non-CNS solid cancer seems to be multifactorial. Although the mechanism underling cognitive alteration is still under investigation, it was hypothesised to be related to neuroimmune and neuroinflammatory changes due to tumour biology and cancer treatments, mainly chemotherapy, and also to other patient characteristics like age, cognitive reserve, genetic risk factors, comorbid conditions, and chronic medication [6,24]. We demonstrated a positive association between PCI, advanced age, and the presence of polypharmacy. 

Even if the PCI pattern varies across patients and cancer type or staging, it is most commonly expressed by variable degrees of memory, executive functions and processing speed alterations [25,26]. In our study, we found that Mini-Cog test, used for cognitive function evaluation, is easy to apply and well accepted by the patients. Comparing with other largely utilised cognitive function evaluation tools, such as Mini Mental State Examination (MMSE), Mini-Cog test takes a shorter amount of time for administration and scoring (2–5 min versus 7–10 min) and had increased sensitivity (99% vs. 91%) and specificity (92% vs. 93%) to detect cognitive impairment [27,28]. 

Our results, showing that more than half of elderly surgical cancer patients experienced PCI, compare well with previous studies performed in general oncologic or in geriatric surgical patients. Some data showed that almost 40% of oncologic patients can develop PCI prior to any cancer treatment [26,29]. Wefel et al. showed that 33% of breast cancer women presented cognitive impairment prior to any systemic therapy [30]. This has been shown in other types of cancer as well. Vardy et al. demonstrated that 43% of colorectal cancer patients had impairment on neurocognitive testing before chemotherapy compared with 15% of healthy controls [31] and Yao et al. showed evidence of executive dysfunction in breast cancer patients prior to either surgery or chemotherapy [32]. Huisman et al. demonstrated a 34.5% prevalence of cognitive impairment (screened with MMSE and defined as a MMSE score > 26) in elderly surgical cancer patients [33]. Recently published preliminary results of a multicentric international GOSAFE (Geriatric Oncology Surgical Assessment and Functional rEcovery after Surgery) study that enrolled 977 patients showed a 20.9% prevalence of preoperative cognitive impairment diagnosed by a Mini-Cog >2 [34]. Approximately two-thirds of patients in our study were diagnosed with PCI of mild severity, in line with previously published data [25,26]. It is difficult to clinically detect mild cognitive changes without the use of a standardised screening scale. Its high prevalence underscores the major importance of the preoperative screening process in older cancer patients. 

The literature reports of POD prevalence in elderly cancer patients are variable depending on the screening test and also on patient, primary disease, and surgical procedure characteristics. We selected Nu-DESC test for POD detection as it had a reported sensitivity between 32 and 95% and specificity up to 87%, being the most sensitive test in recovery room [12,19,20,35]. In the present study, using a cut-off Nu-DESC score >2, we detected a 20% POD prevalence in all patients and almost 30% in patients with PCI. This rate was similar with 21% POD prevalence reported by Raats JW. et al., using Delirium Observation Screening Scale [36], but higher than 14.1% reported by Tei et al. using Confusion Assessment Method [37] and 12.3% demonstrated by Monacelli et al. using experienced geriatrician evaluation based on DSM-V criteria [38], all in colorectal elderly cancer patients. 

We also analysed the relationship between preoperative PCI and POD development. Previously published data in non-oncologic patients showed that cognitive impairment is a risk factor for POD [7,39,40]. We proved that elderly cancer patients with a preoperative Mini-Cog score 0–3 are more prone to develop delirium during the postoperative period. The results of our study reinforce the recommendation that clinicians involved in the perioperative management of older cancer patient should screen for cognitive impairment based on the proved positive prediction for POD.

The main limitations of this study were as follows: it was carried out at a single institution and the intraoperative cerebral function monitoring during general anaesthesia was not available in all patients. Additionally, preoperative patients’ frailty was partially assessed and not recorded, and the period of time for POD assessment (48 h) was relatively short. 

To our knowledge, the current study is the first to analyse the prevalence of both PCI and POD in elderly oncological patients with various types of solid tumours.

Based on the high prevalence of perioperative neurocognitive disorders identified in the present study, we are planning future research projects addressing POD risk reducing strategies. We plan to perform cognitive prehabilitation and tailored intraoperative and postoperative management including opioid-sparing multimodal analgesia protocol, minimisation of high risk medication, optimal pain control, and introduction of nonpharmacologic interventions to prevent POD.

## 5. Conclusions

In a cohort of elderly cancer patients, we demonstrate that Mini-Cog, an easy and fast performed cognitive screening test, can both identify preoperative PCI and predict which patients are most likely to develop POD. PCI is highly prevalent among onco-geriatric patients and screening for cognitive impairment should become a routine part of the preoperative evaluation. A Mini-Cog score equal or less than 3 helps to identify a subgroup of cancer patients at risk for delirium.

## Figures and Tables

**Table 1:** Preoperative variables in elderly cancer patients according to Mini-Cog score.

Characteristics	Total Group (*n* = 131)	Mini-Cog Score ≤ 3 (*n* = 68)	Mini-Cog Score ≥ 4 (*n* = 63)	*p*-Value
Age, years (mean ± SD) ^§^ median (range)	72.1 ± 5.9 71 (67; 76)	74 ± 6.3 74 (68; 79.5)	70 ± 4.7 69 (66; 72)	0.001 *
Gender (male/female), *n* (%) ^†^	66/65 (49.6/50.4)	29/39 (42.7/57.3)	37/26 (58.7/41.3)	0.065
BMI, kg/m² (mean ± SD) ^§^ median (range)	25.7 ± 3.9 25 (23; 29)	25.8 ± 3.9 25 (23; 29)	25.6 ± 4 25 (24; 28)	0.789
Education level, *n* (%) ^†^				
College graduate	18 (13.7)	8 (11.8)	10 (15.9)	0.541
High school	72 (55)	36 (52.9)	36 (57.2)	
Less than high school	41 (31.3)	24 (35.3)	17 (26.9)	
Alcohol consumption, *n* (%) ^†^	45 (34)	22 (32.4)	23 (36.5)	0.617
Sensorial deficits, *n* (%) ^†^	15 (11.5)	9 (13.2)	6 (9.5)	0.505
Charlson Comorbidities Index, *n* (%) ^†^				
0	13 (9.9)	4 (5.9)	9 (14.3)	0.175
1	34 (19.8)	18 (26.5)	16 (25.4)	
2	26 (26)	11 (16.8)	15 (23.8)	
≥3	58 (44.3)	35 (51.5)	23 (36.5)	
Polypharmacy, *n* (%) ^†^	76 (58)	45 (66.2)	31 (49.2)	0.036 *
ASA Score, *n* (%) ^†^				
II	64 (48.9)	32 (47.1)	32 (50.8)	0.463
III	67 (51.1)	36 (52.9)	31 (49.2)	
Type of cancer, *n* (%) ^†^				
Oesophageal, gastric	10 (7.6)	2 (2.9)	8 (12.7)	0.581
Colorectal	40 (30.5)	23 (33.8)	17 (27)	
Liver, gallbladder, pancreatic	12 (9.2)	6 (8.8)	6 (9.5)	
Breast	12 (9.2)	6 (8.8)	6 (9.5)	
Gynaecologic	21 (16)	12 (17.6)	9 (14.3)	
Lung	7 (5.3)	3 (4.4)	4 (6.3)	
Renal, prostate, bladder	16 (12.2)	10 (14.7)	6 (9.5)	
Skin, soft tissue	13 (9.9)	6 (8.8)	7 (11.1)	

^§^ Mann–Whitney U test; ^†^ Pearson Chi-square test; SD—standard deviation; BMI—body mass index; ASA—American Society of Anaesthesiologists; (*) Marked effects are significant at *p* < 0.05.

**Table 2:** Perioperative variables in elderly cancer patients according to Nu-DESC score.

Characteristics	Total Group (*n* = 131)	Nu-DESC ≥ 2 (*n* = 26)	Nu-DESC < 2 (*n* = 105)	*p*-Value
Age, years (mean ± SD) ^§^ median (range)	72.1 ± 5.9 71 (67; 76)	74.7 ± 5.8 73.5 (70; 80)	71.6 ± 5.8 70 (67; 75)	0.007 *
Gender (male/female), *n* (%) ^†^	66/65 (49.6/50.4)	14/12 (46.2/53.8)	54/51 (51.4/48.6)	0.629
BMI, kg/m² (mean ± SD) ^§^ median (range)	25.7 ± 3.9 25 (23; 29)	24.5 ± 4.3 24.5 (22; 27)	26 ± 3.8 25 (24; 29)	0.072
Education level, *n* (%) ^†^				
College graduate	18 (13.7)	4 (15.4)	14 (13.3)	0.587
High school	72 (55)	16 (61.5)	56 (53.3)	
Less than high school	41 (31.3)	6 (23.1)	35 (33.3)	
Alcohol consumption, *n* (%) ^†^	45 (34.4)	7 (26.9)	38 (36.1)	0.365
Sensorial deficits, *n* (%) ^†^	15 (11.5)	3 (11.4)	12 (11.5)	0.987
Charlson Comorbidities Index, *n* (%) ^†^				
0	13 (9.9)	1 (3.8)	12 (11.4)	0.215
1	34 (25.9)	4 (15.4)	30 (28.6)	
2	26 (19.9)	7 (26.9)	19 (18.1)	
≥3	58 (44.3)	14 (53.9)	44 (41.9)	
Polypharmacy, *n* (%) ^†^	76 (58)	14 (53.9)	62 (59.1)	0.631
ASA Score, *n* (%) ^†^				
II	64 (49)	8(30.7)	56 (53.3)	0.037 *
III	67 (51)	18(69.2)	49 (46.6)	
Type of anaesthesia, *n* (%) ^†^				
General anaesthesia	113 (86.3)	26 (100)	87 (82.9)	0.023 *
Loco-regional anaesthesia	18 (13.7)	0	18 (17.1)	
Type of cancer, *n* (%) ^†^				
Oesophageal, gastric	10 (7.6)	3 (11.5)	7 (6.6)	0.038 *
Colorectal	40 (30.5)	11 (42.3)	29 (27.6)	
Liver, gallbladder, pancreatic	12 (9.2)	3 (11.5)	9 (8.5)	
Breast	12 (9.2)	0	12 (11.4)	
Gynaecologic	21 (16)	5 (19.2)	16 (15.2)	
Lung	7 (5.3)	1 (3.8)	6 (5.7)	
Renal, prostate, bladder	16 (12.2)	3 (11.5)	13 (12.4)	
Skin, soft tissue	13 (9.9)	0	13 (12.4)	

^§^ Mann–Whitney U test; ^†^ Pearson Chi-square test; SD—standard deviation; Nu-DESC—Nursing Delirium Screening Scale; BMI—Body mass index; ASA—American Society of Anaesthesiologists; (*) Marked effects are significant at *p* < 0.05.

**Table 3:** Sensitivity and specificity of Mini-Cog test in predicting postoperative delirium.

Mini-Cog Score	Sensitivity	95% CI	Specificity	95% CI	PPV	NPV
0	0.1923	0.0655–0.3935	0.9540	0.8864–0.9873	0.8070	0.5415
1	0.3462	0.1721–0.5567	0.8966	0.8127–0.9516	0.7700	0.5783
2	0.5000	0.2993–0.7007	0.6897	0.5814–0.7845	0.6171	0.5797
Cut-off 3 (*p* = 0.006)	0.7308	0.5221–0.8843	0.5172	0.4075–0.6258	0.6022	0.6577
4–5	0.8846	0.6985–0.9755	0.3448	0.2461–0.4544	0.5745	0.7492

CI—confidence interval, PPV—positive predictive value, NPV—negative predictive value.

**Table 4:** Predicted probability of postoperative delirium according to Mini-Cog score (based on Joinpoint regression model).

Segment	The Edges of the Segment: Mini-Cog Score		APC	APC		Test Statistic (t)	*p*-Value
	Lower Endpoint	Upper Endpoint		Lower 95%CI	Upper 95%CI		
**Full Range**	**0**	**5**	−27.41	−40.31	−11.71	−4.54	0.01

APC—percentage change in POD probability (%) for a one-unit increase in Mini-Cog score; CI—confidence interval.

**Table 5:** Age-adjusted univariate and multivariate logistic regression analyses to identify predictors for post-operative delirium.

Post-Operative Delirium Age-Adjusted vs.	Univariate Analysis		Multivariate Analysis	
	Adjusted OR (95% CI)	*p*-Value	Adjusted OR (95% CI)	*p*-Value
Mini-Cog score (≤3)	2.691 (1.022–7.084)	0.006 *	2.630 (1.153–3.991)	0.027 *
Clock draw	2.963 (1.176–7.462)	0.021 *	2.909 (1.723–9.684)	0.013 *
Word recall	1.619 (1.112–4.283)	0.032 *	1.125 (1.052–2.362)	0.061 *
Gender (female)	1.215 (0.498–2.960)	0.669		
BMI	0.927 (0.826–1.041)	0.203		
Education (no College graduate)	1.638 (0.592–4.534)	0.342		
ASA score (≤2)	1.552 (0.461–4.781)	0.474		
Charlson Comorbidities Index	1.576 (0.960–2.588)	0.072		
Alcohol consumption	1.330 (0.500–3.537)	0.568		
Preoperative anaemia	1.006 (0.541–1.870)	0.984		
Preoperative medication	1.466 (0.599–3.585)	0.402		
Sensorial deficits	1.064 (0.269–4.204)	0.930		
Preoperative renal function	3.218 (1.254–8.259)	0.015 *	2.625 (1.854–8.066)	0.012 *
Type of surgery	2.681 (1.489–4.949)	0.023 *	0.692 (0.448–1.068)	0.096
Ketamine	0.625 (1.256–1.530)	0.304		
Morphine	3.352 (1.317–8.535)	0.011 *	2.727 (1.895–8.303)	0.007 *
Metoclopramide	5.864 (4.922–10.271)	0.041 *	6.631 (2.823–13.436)	0.006 *
Surgery duration	1.505 (1.267–3.518)	0.032 *	0.801 (0.286–2.240)	0.672
Postoperative pain score (NRS)	1.311 (1.056–1.629)	0.014 *	1.861 (1.070–2.640)	0.018 *

OR—odd ratio; CI—confidence interval; BMI—body mass index, ASA—American Society of Anaesthesiology, NRS—Numeric Rating Scale; (*) Marked effects are significant at *p* < 0.05.

**Figure A1:**
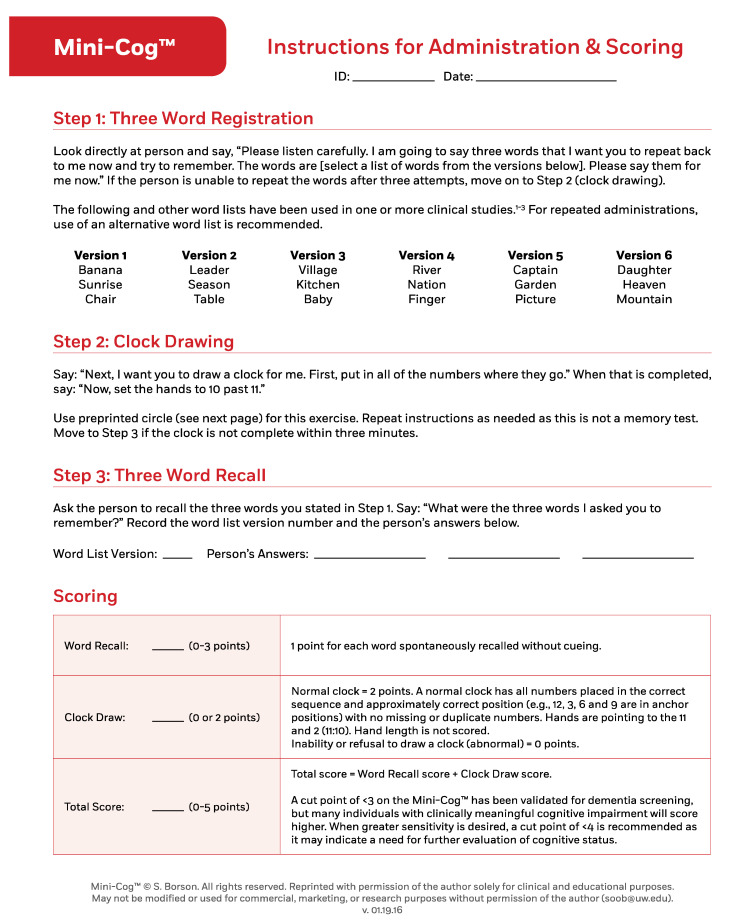
The Mini‒Cog test.

**Figure A2:**
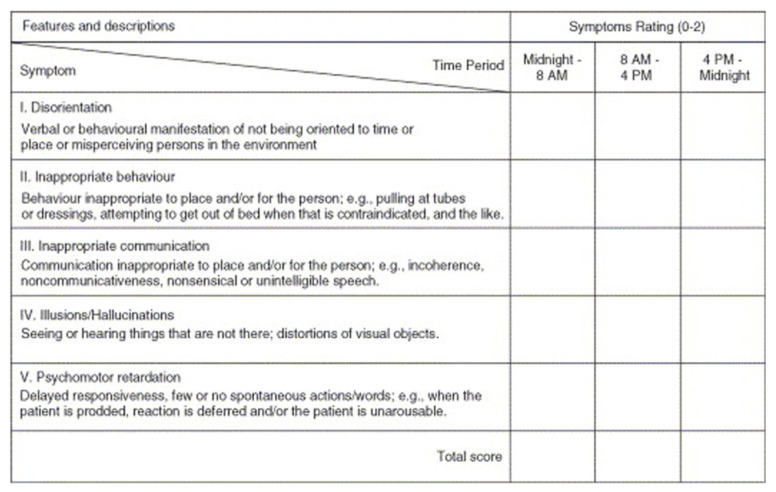
The Nu-DESC score.
